# Reliability of a participant-friendly fecal collection method for microbiome analyses: a step towards large sample size investigation

**DOI:** 10.1186/s12866-018-1249-x

**Published:** 2018-09-06

**Authors:** Joanna W. Szopinska, Raphaële Gresse, Saskia van der Marel, Jos Boekhorst, Sabina Lukovac, Iris van Swam, Barbara Franke, Harro Timmerman, Clara Belzer, Alejandro Arias Vasquez

**Affiliations:** 1 Department of Psychiatry, Radboudumc, Donders Institute for Brain, Cognition and Behaviour, P.O. Box 9101 HB, Nijmegen, The Netherlands; 2 UMR 454 MEDIS UCA-INRA, Université Clermont Auvergne, F-63000 Clermont-Ferrand, France; 3 Department of Human Genetics, Radboudumc, Donders Institute for Brain, Cognition and Behaviour, P.O. Box 9101 HB, Nijmegen, The Netherlands; 4 NIZO Food Research BV, P.O. Box 20, 6710 BA Ede, The Netherlands; 5 Laboratory of Microbiology, Wageningen University, Stippeneng 4, 6708 WE Wageningen, The Netherlands; 6 Department of Cognitive Neuroscience, Radboudumc, Donders Institute for Brain, Cognition and Behaviour, P.O. Box 9101 HB, Nijmegen, The Netherlands

**Keywords:** Microbiome, OMNIgene•GUT, Bacterial DNA extraction, Next generation sequencing, Fecal collection and storage method

## Abstract

**Background:**

The effects of gut microbiota on human traits are expected to be small to moderate and adding the complexity of the human diseases, microbiome research demands big sample sizes. Fecal samples for such studies are mostly self-collected by participants at home. This imposes an extra level of complexity as sample collection and storage can be challenging. Effective, low-burden collection and storage methods allowing fecal samples to be transported properly and ensuring optimal quality and quantity of bacterial DNA for upstream analyses are necessary. Moreover, accurate assessment of the microbiome composition also depends on bacterial DNA extraction method. The aim of this study was to evaluate the reliability and efficiency of the OMNIgene•GUT kit as a participant-fecal friendly collection method (storage at room temperature for 24 h (O24h) or 7 days (O7d)) in comparison to the standard collection method (Fresh, storage at 4 °C for less than 24 h) in terms of amount of variability and information content accounting for two common DNA extraction methods.

**Results:**

Fourteen fecal samples were collected from healthy individuals (7 males, 7 females). Collection and storage methods did not differ significantly in terms of DNA concentration and Shannon diversity index. Phylum relative abundance showed significant differences for Bacteroidetes, Actinobacteria and Cyanobacteria. The differences were observed between control (Fresh) and O24h methods, but not between Fresh and O7d. These differences were not seen when performing bacterial DNA quantification based on three bacterial groups: *Bacteroides* spp., *Bifidobacterium* spp. and *Clostridium* cluster IV, which represent three major phyla: Bacteroidetes, Actinobacteria and Firmicutes respectively. The two DNA extraction methods differ in terms of DNA quantity, quality, bacterial diversity and bacterial relative abundance. Furthermore, principal component analysis revealed differences in microbial structure, which are driven by the DNA extraction methods more than the collection/storage methods.

**Conclusion:**

Our results have highlighted the potential of using the OMNIgene•GUT kit for collection and storage at ambient temperature, which is convenient for studies aiming to collect large samples by giving participants the possibility to send samples by post. Importantly, we revealed that the choice of DNA extraction method have an impact on the microbiome profiling.

**Electronic supplementary material:**

The online version of this article (10.1186/s12866-018-1249-x) contains supplementary material, which is available to authorized users.

## Background

The human gastrointestinal tract houses a highly complex ecosystem composed of ten to one hundred trillion microbial cells called the gut microbiota [1]. Gut microbes play an important role in human health by providing important metabolic, immunological and developmental functions [2, 3]. Recent studies have suggested a link between changes (i.e. dysbiosis) in the human gut microbiota and a wide variety of diseases and syndromes including obesity, irritable bowel syndrome, allergies, liver and skin diseases as well as neurodevelopmental disorders [4–6]. Microbiota-based therapies are now considered a non-pharmacological treatment alternative for several metabolic and immune related disorders [7], and may offer promise suggested for neuropsychiatric disorders [8].

An accurate analysis of the microbiome structure, in relation to human traits and diseases, at the population level, relies on large sample sizes. This facilitates analysis with adequate statistical power and limit the influence of outliers and extreme observations [9–11]. However, current methods of collecting fecal samples demand high involvement from participants to set up appointments on the collection day and storing the samples in their own fridge or freezer, which can bring discomfort for the participants and potentially represent a health risk. Those factors might negatively affect participants’ decision to take part in a study. Most times, immediate freezing needed in the standard collection method may be not feasible [12]. Recently, more elegant methods for sample collection and early storage have been developed. Here, we tested a commercial kit (OMNIgene•GUT) which allows collection and storage of fecal samples at room temperature (RT). Use of such kits enables participants to send their samples via regular post without the need of making appointments, refrigeration, or cold-chain transportation and might increase participation for (future) research studies.

Collection, sample storage and bacterial DNA extraction methods are key steps required for the accuracy of the studies of human intestinal microbiota composition without (minimal) loss of any taxa [13]. If immediate processing of samples is not feasible (i.e. geographical distances or temperature and storage requirements), microbial community representation might be affected. The technical sources of variation (i.e. sample collection and storage techniques, DNA extraction method) have a large influence on the observed structure of the microbial community, often on scales similar to or larger than biological effects [14]. In the present study we tested differences in microbiome profile determined by two different commercial DNA extraction methods, QIAamp DNA Mini Kit (QIA; QIAGEN, Venlo, NL) and PowerFecal® DNA Isolation Kit (PF; MO BIO Laboratories, Inc.).

The aim of the study was a two-fold: (i) to test the OMNIgene•GUT (DNA Genotek, Ottawa, CA) feces collection and storage kit in terms of DNA quality and quantity, bacterial diversity and composition based on the quantification of genes coding for 16S ribosomal RNA (rRNA), and to compare it to the standard collection and storage method (called here the ‘Fresh’ method) and (ii) to determine the collection and storage method while accounting for two common DNA extraction methods, QIAamp DNA Mini Kit (QIA) and PowerFecal® DNA Isolation Kit (PF). We addressed DNA extraction method issue because it is a part of overall efficacy of collection strategy.

## Methods

### Sample collection, storage and DNA isolation

All participants included in this study were adults (18<). Fecal samples were collected from 14 healthy individuals (7 males, 7 females) in triplicate (1× Fresh, 2× OMNIgene•GUT, *N* = 42). The first group of samples collected via the Fresh method was stored at 4 °C right after collection and processed within 24 h. The other two groups of samples were collected using the OMNIgene•GUT kit (DNA Genotek, Ottawa, CA) and kept at room temperature (RT) up to 24 h (O24h) or 7 days (O7d) and then continue with DNA extraction (Additional file 1: Figure S1). The total amount of sample used (from the OMNIgene•GUT kit) for DNA extraction was 0.25 mL containing approximately 50 mg feces and 200 μL stabilizing liquid. DNA was extracted using QIAamp DNA Mini Kit (QIA; QIAGEN, Venlo, NL) according to the manufacturer’s instructions, including bead-beating steps using a FastPrep®-24 Instrument (MP Biomedicals, Amsterdam, NL). To compare the effects of two different DNA extraction methods, DNA from the O24h samples was also isolated using PowerFecal® DNA Isolation Kit (PF; MO BIO Laboratories, Inc.) recommended by DNA Genotek (Ottawa, CA) (Additional file 2: Figure S2). DNA extraction procedures were performed at NIZO food research (Ede, NL).

Given our aim and that we had two DNA extraction approaches we decided to test their efficacy and efficiency on the samples collected with the OMNIgene•GUT kit and stored for 24 h (O24h) because these should, in principle, conserve the bacterial DNA better as those stored for 7 days (O7d). This decision was made prior to the sequencing experiments and analysis. The PF method was tested as recommendation by the manufacturers of the OMNIgene•GUT. The QIA method came as a recommendation from our co-authors from NIZO which have extensive experience with bacterial DNA extraction.

### PCR amplification and sequencing

The PCR amplicons of bacterial 16S rRNA V3-V4 regions were generated using 2-step polymerase chain reaction (PCR). Universal primers with adaptor sequences for use in Illumina MiSeq assays were used for an initial amplification of the V3-V4 part of the 16S rRNA gene with the following sequences: forward primer ‘5-CCTACGGGAGGCAGCAG-3’ (primer 357F); reverse primer ‘5-TACNVGGGTATCTAAKCC’ (adapted 802R). PCR amplification mixture contained: 1 μL of template DNA, 1 μL of the forward primer (10 μM; 357F), 1 μL of the reverse primer (10 μM; 802R), 1 μL KOD Hot Start DNA Polymerase (1 U/μL; Novagen, Madison, WI, USA), 5 μL KOD-buffer (10×), 3 μL MgSO4 (25 mM), 5 μL dNTP mix (2 mM each), and 33 μL nuclease-free water (total volume 50 μL). Reactions were held at 95 °C for 2 min followed by 30 cycles at 95 °C for 20 s, 55 °C for 10 s, and 70 °C for 15 s. The PCR amplicons of approximately 500 bp were subsequently purified using the MSB Spin PCRapace kit (Invitek, STRATEC Molecular GmbH, Berlin, DE). Concentration and quality were subsequently checked by using 2000 spectrophotometer (Thermo Scientific, Breda, NL). Purified PCR products were shipped to BaseClear BV (Leiden, NL) and used for the second PCR in combination with sample-specific barcoded primers. DNA was used for PCR amplification at a concentration of 8 ng/μL. PCR products were purified using the Mini Elute PCR Purification kit (QIAGEN, Venlo, NL). Amplicon libraries were normalized based on their ng/μL concentration, after which the pM concentration of the pool was determined using the Kapa Illumina library Quantification kit. Final loading concentration of the libraries on the MiSeq was targeted at 5.5 pM with a 10% PhiX spike. All samples were processed and prepped at the same time and ran on a single Illumina lane. Sequencing of libraries was performed with Illumina MiSeq platform with the paired-end (2×) 300 bp protocol. We used FASTQC and the Illumina CASAVA pipeline (v1.8.3) to achieve high-quality sequences as described in PMID28774885 [15].

### Processing of sequencing data and statistical analysis

Read pairs were assembled into pseudoreads with PEAR, using the default settings [16]. Sequence data was analyzed using a workflow based on the software tool QIIME (Quantitative Insights Into Microbial Ecology) version 1.8 [17]. Reference-based chimera removal was done using UCHIME as implemented in QIIME 1.8. OTUs consisting of only a single sequence were removed. Sequences that could not be aligned by PyNAST against the 16S reference alignment were removed. Operational taxonomy units (OTU) clustering (open reference), taxonomic assignment and reference alignment was done with the ‘pick_open_reference_otus.py’ workflow script of QIIME, using ‘UCLUST’ as clustering method (97% identity) and Greengenes version 13.8 as a reference [18]. Sequence depth per sample was investigated by comparing the read counts between three different collection methods and two different DNA extraction methods. Alpha diversity was calculated using the ‘alpha_rarefaction.py’ workflow script. Reference-based chimera removal was done with UCHIME [19]. The Ribosomal Database Project (RDP) classifier version 2.2 was performed for taxonomic classification. In order to compare samples to each other we transform the read counts into relative abundance (normalization step). Taxa-specific read counts were divided by the total number of reads for that sample to which taxa belongs in order to normalized them. The resulting normalized read counts for each taxon represented a measure of the relative abundance of the various taxa identified in that sample. The sum of the taxa relative abundance per sample is 100%. Differences in microbiota composition were determined using paired t-test or Wilcoxon signed-rank test, following by Shapiro-Wilk test for normality and inspected visually by a histogram. The statistical analyses were performed using SPSS v.22.0. Bonferroni correction for multiple comparisons was applied accordingly. The variation in the data was visualized with principal component analysis (PCA) and done in Canoco 5.0.4 [20]. Through all study we applied four comparisons: (i) Fresh+QIA vs. O24h + QIA, (ii) Fresh+QIA vs. O7d + QIA, (iii) O24h + QIA vs. O7d + QIA and (iv) O24h + QIA vs. O24h + PF.

### Bacterial genomic DNA

In order to confirm observed results from sequencing analysis, we quantified three bacterial groups: *Bacteroides* spp., *Bifidobacterium* spp. and *Clostridium* cluster IV belonging to the three major phyla Bacteroidetes*,* Actinobacteria and Firmicutes, respectively. Standard for the absolute quantification of these three genera was obtained using genomic DNA of *Bacteroides fragilis* (ATCC 10584) and *Bifidobacterium adolescentis* (ATCC 15703) grown at the Department of Medical Microbiology of Radboudumc (Nijmegen, NL) as well as *Clostridium leptum* (ATCC 753) provided by Deutsche Sammlung von Mikroorganismen und Zellkulturen GmbH (DSMZ; Braunschweig, DE).

In order to detect and quantify *Bacteroides* spp., *Bifidobacterium* spp., and *Clostridium* cluster IV, the group-specific primers based on 16S rDNA sequences and probes were used. The probes, primers, and expected amplicon sizes for PCR are summarized in Table 1.

**Table 1:** Primers and probes used to carry out real time PCR quantification of *Bacteroides* spp., *Bifidobacterium* spp. and *Clostridium* cluster IV

Target	Name	Sequence	Amplicon Length	Concentration nM	Ref.
Clostridium cluster IV	sg-Clep-F	GCA CAA GCA GTG GAG T	239	400	[29]
	sg-Clep-R	CTT CCT CCG TTT TGT CAA		400	
	Clep-Pa	(FAM)-AGG GTT GCG CTC GTT-(TAMRA)		200	
Bifidobacterium spp.	F-bifido	CGCGTCYGGTGTGAAAG	244	300	[30]
	R-bifido	CCCCACATCCAGCATCCA		300	
	MGB-bifido	(FAM)-AACAGGATTAGATACCC-(MGB)		200	
Bacteroides spp.	AllBac296f	GAG AGG AAG GTC CCC CAC	106	400	[31]
	AllBac412r	CGC TAC TTG GCT GGT TCA G		400	
	AllBac375Bhqr	(FAM)-CCA TTG ACC AAT ATT CCT CAC TGC TGC CT-(TAMRA)		200	

*Target* Selected species (spp.) tested with qPCR, *Name* Name of primers and probes used to amplify Target, *Sequence* primers/probes sequence, *Amplicon length* the PCR product size of the Target in base pars (bp), *Concentration nM* concentration of the primers/probes needed to perform experiment, *nM* nanomolar, *Ref.* references where the method is described

### Construction of standard curves for 16S rRNA gene copy number determination

In order to have an adequate number of DNA fragments for downstream analysis, conventional PCR for the amplification of the 16S rRNA genes was carried out on a genomic DNA of bacterial strains described above. The reaction was performed using the universal bacterial primers (i) for *Bacteroides* spp. and *Clostridium* cluster IV 27F (5’-AGAGTTTGATCCTGGCTCAG-3′) and 1492R (5’-CGGCTACCTTGTTACGAC-3′) and (ii) for *Bifidobacterium* spp. 388F (5’-ACTCCTACGGGAGGCAGCAG-3′) and 1492R. The PCR mixture (50 μL) was composed of 10× PCR buffer II (Applied Biosystems, Foster City, California, USA), 2.5 mM MgCl_2_, each dNTP (deoxynucleoside triphosphate) at a concentration of 2.5 mM, each primer at a concentration of 50 μM, 10–100 ng genomic DNA and 5 units/μL of Taq DNA polymerase (TAKARA Bio Inc., Kusatsu, Shiga, JPN). The PCR was carried out on an iCycler thermal cycler (Bio-Rad, Hercules, California, USA) under the following conditions: 94 °C for 5 min, 35 cycles of 94 °C for 30 s, 52 °C for 40 s, and 72 °C for 90 s; and finally 72 °C for 7 min. The PCR products were purified using the Mini Elute PCR Purification kit (QIAGEN, Venlo, NL) according to the manufacturer’s instructions and were subjected after purification to a 1% agarose gel electrophoresis containing GelRed and visualized for being (approximately) 1465 bp in size. DNA quantity and quality was measured via NanoDrop 2000 spectrophotometer (Thermo Scientific, Breda, NL).

### Real-time PCR assay

The purified PCR products of aforementioned bacterial strains were used as standard-samples in real-time PCR (qPCR) in order to calculate the 16S gene copy number. qPCR and Next Generation Sequencing (NGS) appear as robust and efficient tools for the detection and/or quantification of a wide range of bacterial DNA. Therefore, qPCR was used to confirm observed results from the sequencing analysis. The assay was performed with an ABI PRISM 7900HT sequence detection system (Applied Biosystems, Foster City, California, USA) in a volume of 10 μL with TaqMan® Fast Universal PCR Mastermix (Applied Biosystems, Foster City, California, USA) with the addition of each primer and probe at specific concentrations described in Table 1. The program cycle conditions were: 50 °C for 2 min and 95 °C for 10 min, followed by (i) for *Bacteroides* spp. 40 cycles of 95 °C for 30 s, 60 °C for 1 min and 72 °C for 1 min (ii) for *Bifidobacterium* spp. 40 cycles of 95 °C for 15 s, 60 °C for 1 min and 72 °C for 1 min and (iii) for *Clostridium* cluster IV 40 cycles of 95 °C for 30 s, 53 °C for 45 s, and 60 °C for 1 min. The 16S rRNA gene copy number was calculated using the equation:

$$ \mathbf{copy}\ \mathbf{number}/\boldsymbol{\upmu} \mathbf{L}=\left(\mathbf{C}/\mathbf{X}\right)\mathbf{x}\mathbf{0.912}\mathbf{x}\mathbf{1012} $$

where C: DNA concentration measured (ng/μL) and X: PCR fragment length (bp/copy) [21].

### Calculation of log 16S gene copy number per gram of feces

In order to quantify aforementioned bacterial DNA, we calculated the log 16S gene copy number per gram of faces. By taking into account the threshold cycle (C_T_) values obtained by a known concentration of 16S rRNA, the intercept, the slope, the DNA extraction volume, the sample dilution factor, the fecal sample weight and the number of μL per well.

## Results

In the present study, (i) we compared two collection and storage methods (Fresh; OMNIgene•GUT) and (ii) we accounted for two common DNA extraction methods (QIA; PF) experimental design can be found in Additional file 1: Figure S1 and Additional file 2: Figure S2, respectively.

### DNA concentration and purity

OMNIgene•GUT kit (O24h; mean concentration = 267.22 ng/uL; *N* = 14) had a lower DNA concentration than Fresh (mean concentration = 304.60 ng/uL; N = 14), although those results were not statistically significant (paired t-test; *P* > 0.05). The DNA concentration of the samples collected with the OMNIgene•GUT kit after 7 days of storage (at room temperature; O7d) had lower values (mean concentration = 223.27 ng/uL; N = 14; *P* > 0.05) compared to O24h (Table 2). Furthermore, O7d and Fresh also did not show differences, (*P* > 0.05). DNA purity of the Fresh, O24h and O7d showed similar optimal values for the ratio of absorbance and those values did not show statistical differences (*P* > 0.05) (Table 2).

**Table 2:** DNA quantification and purification values. Mean values with standard deviation (SD) for DNA concentration and purity of fecal samples collected and stored by three different collection/storage methods (Fresh, O24h, O7d) and extracted using two different procedures (PF, QIA)

	NanoDrop measurements (mean ± SD)		
	DNA concentration, ng/μL	260 nm/280 nm ratio	260 nm/230 nm ratio
Fresh + QIA	304.60 ± 106.35	1.86 ± 0.02	1.67 ± 0.20
O24h + QIA	267.22 ± 98.38^a^	1.88 ± 0.01	1.66 ± 0.16^b^
O7d + QIA	223.27 ± 100.03	1.88 ± 0.02	1.60 ± 0.19
O24h + PF	41.31 ± 32.52^a^	2.09 ± 0.39	1.02 ± 0.50^b^

The ratios of absorbance at 260 nm/280 nm and 260 nm/230 nm are used to assess the purity of DNA. A ratio of ~ 1.8 is generally accepted as “pure” for DNA. If the ratio is appreciable lower, it may indicate the presence of contaminants (e.g. proteins, phenols or carbohydrates) (13, 14). ^a^Is the comparison of DNA concentration (column labeled DNA concentration, ng/μL) and ^b^of DNA purity (column labeled 260 nm/230 nm ratio) between QIA and PF DNA extraction methods (Bonferroni-adjusted paired sample t-test with *P* < 0.001 for all tests)

Comparison of the QIA and PF DNA-isolation methods showed differences where PF resulted in approximately seven times lower DNA concentration (PF = 41.31 ng/uL; QIA = 267.22 ng/uL; *P* < 0.001) and about two times lower DNA purity indicated by 260 nm/230 nm ratio (PF = 1.02; QIA = 1.66; *P* < 0.001) (Table 2). This suggests that the QIA method is superior with regard to both DNA concentration and purity.

### Illumina sequencing

16S rRNA gene (16S) sequencing was performed for 56 samples (Additional file 2: Figure S2) and 5 positive controls. After the quality control step it yielded 3,532,503 reads associated to 78,470 OTUs and 49,388 observed bacterial species determined by 16S sequences having 97% similarity. The total read counts per sample were compared between three different collection methods (Fresh, O24h, O7d) and between two different DNA extraction methods to investigate if sequence depth differ between them. No statistically significant differences between all tested methods were found (Additional file 3: Table S1–S2).

### Microbiome composition of the fecal samples

The gut microbiota composition of the participants was mainly characterized by the following phyla: Actinobacteria, Bacteroidetes, Firmicutes, Proteobacteria, and Cyanobacteria (data not shown). We tested whether the microbiome composition of the fecal samples differed among collection/storage methods or DNA extraction kits.

### Alpha diversity

**Fig. 1:**
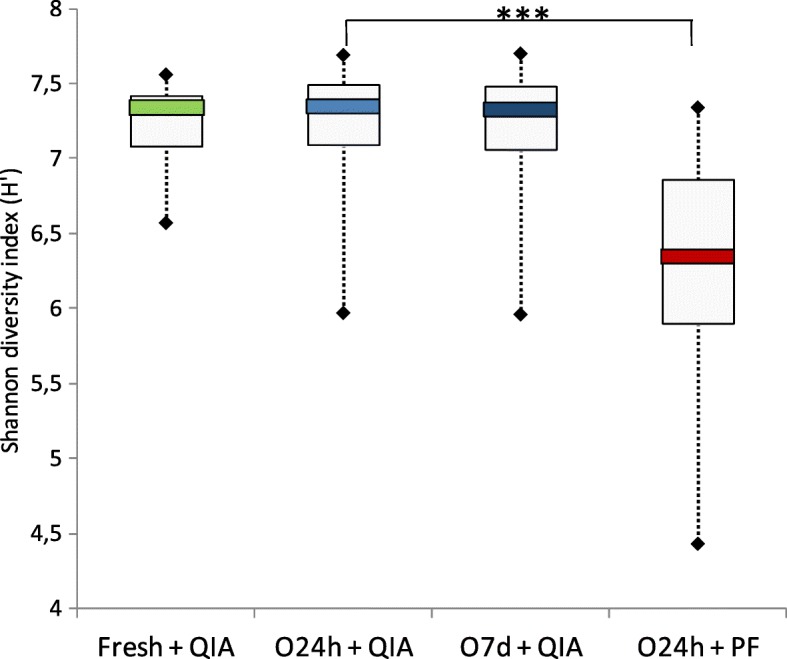
Shannon diversity index of fecal samples collected/stored with three different collection and storage methods (Fresh, O24h, O7d) and extracted using two different procedures (PF, QIA) (***P > 0.001); results determined by sequencing analysis

The impact of the different conditions on the alpha bacterial diversity of the fecal samples was assessed based on the Shannon diversity index. DNA extraction using the PF kit yielded significantly lower alpha diversity scores (within sample variation) compared to the QIA kit (Wilcoxon signed-rank test; *P* > 0.001), whereas no differences were found between collection methods (O24h, O7d, Fresh) (Fig. 1).

### Phylum relative abundance

**Fig. 2:**
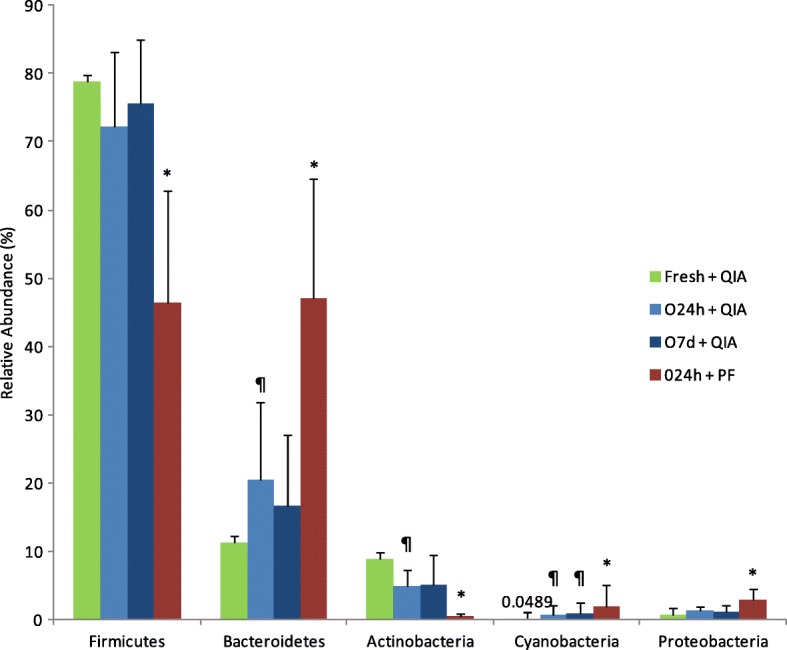
Relative abundance of bacterial taxa at the phylum level in fecal samples collected/stored with three different methods (Fresh, O24h, O7d) and extracted using two different procedures (PF, QIA); results determine by sequencing analysis. Statistical comparisons were performed based on the paired sample t-test. * red bar statistically different from light-blue bar (*P* < 0.05); ¶ blue bar statistically different from green bar (*P* < 0.05)

Samples collected with OMNIgene•GUT (O24h & O7d) showed a trend towards higher phylum relative abundance of Bacteroidetes, Cyanobacteria, Proteobacteria and lower of Firmicutes and Actinobacteria compared to the Fresh method (Fig. 2 and Table 3). Bacteroidetes, Actinobacteria and Cyanobacteria are significantly different (Wilcoxon signed-ranks test; *P* < 0.05; Bonferroni-adjusted) in O24h compared to Fresh. However, the Bonferroni-adjusted *p*-value did not reach statistical significance for O7d compared to Fresh, except for Cyanobacteria (*p* = 0.045). Moreover, phylum relative abundance did not significantly differ across different storage durations used for the OMNIgene•GUT kit (O24h vs. O7d) (Fig. 2 and Table 3).

**Table 3:** Phylum relative abundance of each samples extracted using two different DNA extraction methods and collected with three different procedures; results determined by sequencing analysis

	Mean of phyla relative abundance (%) ± standard error of the mean				
Collection + DNA extraction methods	Firmicutes	Bacteroidetes	Actinobacteria	Cyanobacteria	Proteobacteria
Fresh + QIA	77.82 ± 7.22	11.25 ± 6.23	8.90 ± 4.28	0.05 ± 0.09	0.72 ± 0.46
O24h + QIA	72.10 ± 11.09	20.52 ± 11.39	4.89 ± 2.38	0.74 ± 1.32	1.26 ± 0.76
O7d + QIA	75.52 ± 9.43	16.63 ± 10.46	5.20 ± 4.26	0.90 ± 1.67	1.20 ± 0.83
O24h + PF	46.52 ± 16.21	46.94 ± 17.71	0.54 ± 0.42	1.95 ± 3.12	2.94 ± 1.49

First column refers to the tested collection/storage and DNA extraction methods in the paper. The other columns represent relative abundance of the phyla that were investigated

**Fig. 3:**
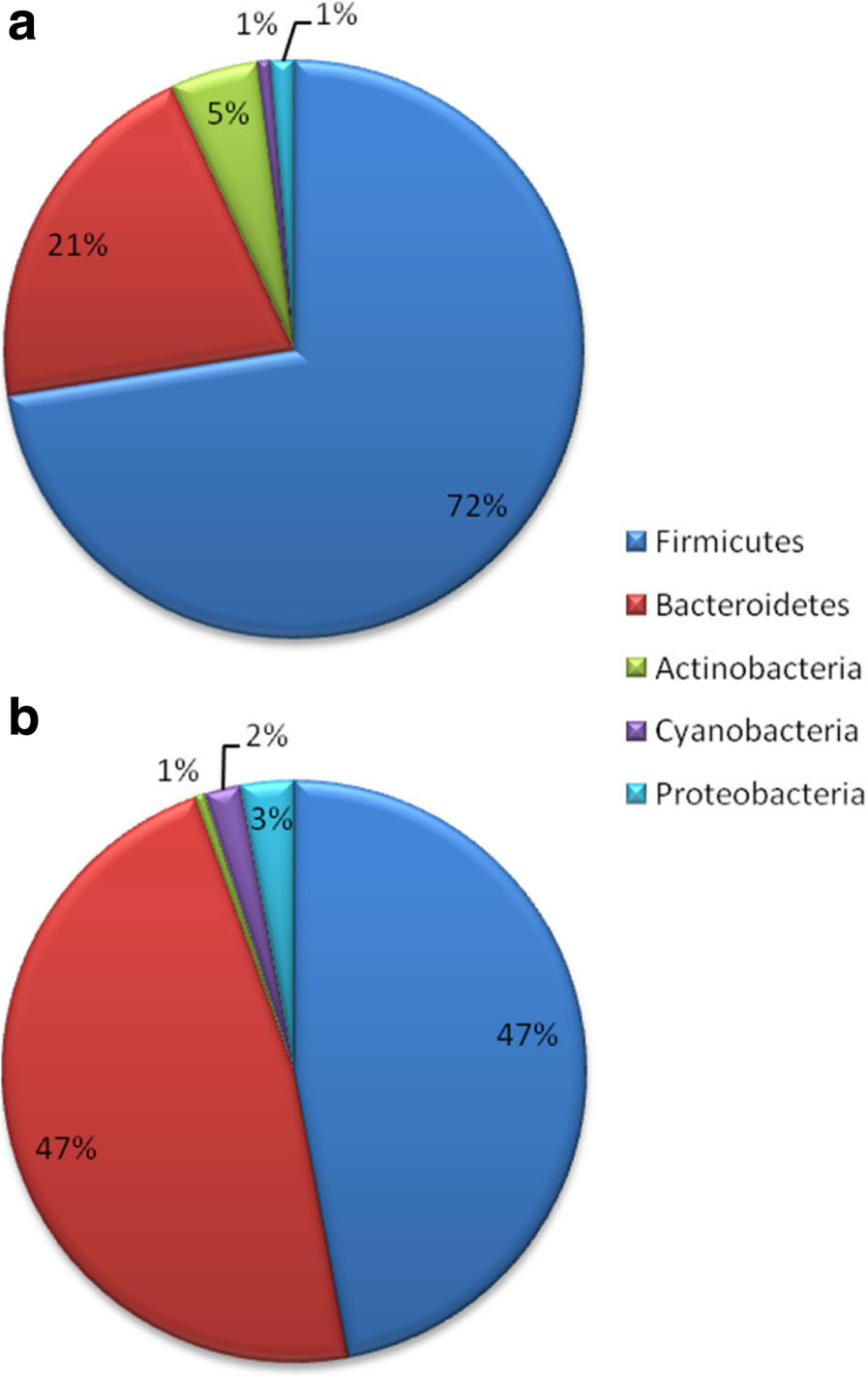
Relative abundance of bacterial phyla of O24h samples treated with QIA (**a**) and PF (**b**) DNA extraction kits; results determined by sequencing analysis. All phyla showed statistically significant differences between two DNA extraction methods (Bonferroni-adjusted Wilcoxon signed-rank test; *P* < 0.05 for all tests)

Relative abundance of the all bacterial phyla (Firmicutes, Bacteroidetes, Actinobacteria, Cyanobacteria and Proteobacteria) differed among DNA extraction methods (Figs. 2 & 3). The most abundant phylum among the O24h samples extracted with QIA was Firmicutes with the average of 72% (Fig. 3a). However, this was not observed in the case of the samples extracted with PF, where Firmicutes average was 47% (Fig. 3b). Compared to QIA, PF showed significantly higher abundance of Bacteroidetes (Bonferroni-adjusted Wilcoxon signed-ranks test; *P* < 0.01), Cyanobacteria (*P* < 0.05) and Proteobacteria (*P* < 0.01), and lower abundance of Firmicutes (*P* < 0.01) and Actinobacteria (*P* < 0.01).

### Microbiome ordination

**Fig. 4:**
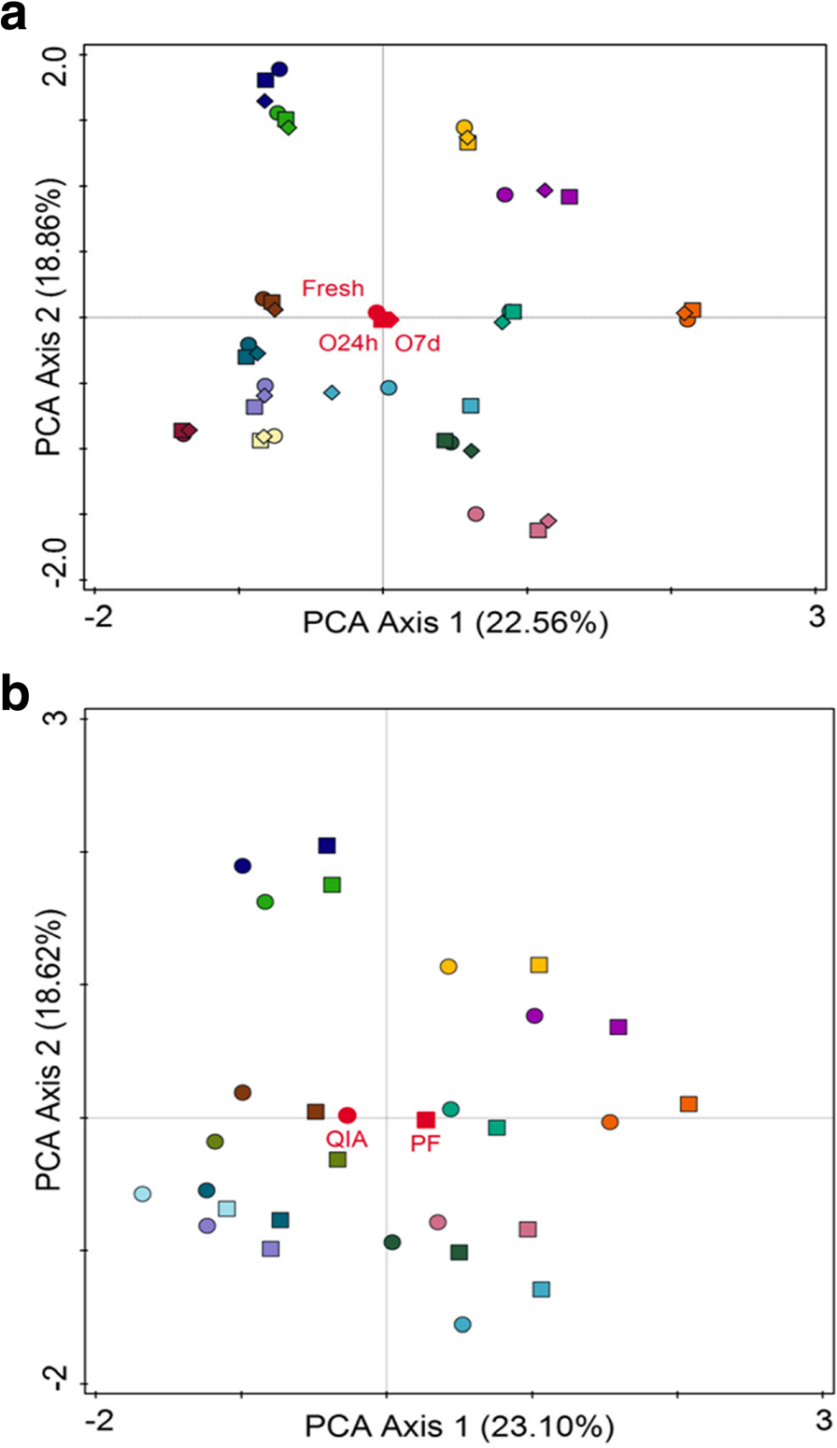
PCA (Principal Component Analysis) plots of bacterial genera relative abundance of 14 individual faecal samples clustered regarding collection and storage methods (Fresh, O24h, O7d) (**a**) or DNA extraction methods (PF, QIA) (**b**). The first two components explained 22.56% and 18.86% of the variance, respectively (**A**) and 23.10% and 18.62% of the variance, respectively (**b**). Faecal collection methods are represented by circle (●) for Fresh, square (■) for O24h, and triangle (▲) for O7d (**a**). DNA extraction methods are represented by circle (●) for QIA, and square (■) for PF (**b**); results determined by sequencing analysis

Principal Component Analysis (PCA) shows that samples belonging to the same individual strongly clustered for 12 out of 14 individuals, across the different collection and storage methods (Fig. 4a). Additionally, collection and storage methods grouped closely together, while the two different DNA extraction methods appeared to cluster less well (Fig. 4b).

### Real-time PCR assay

**Fig. 5:**
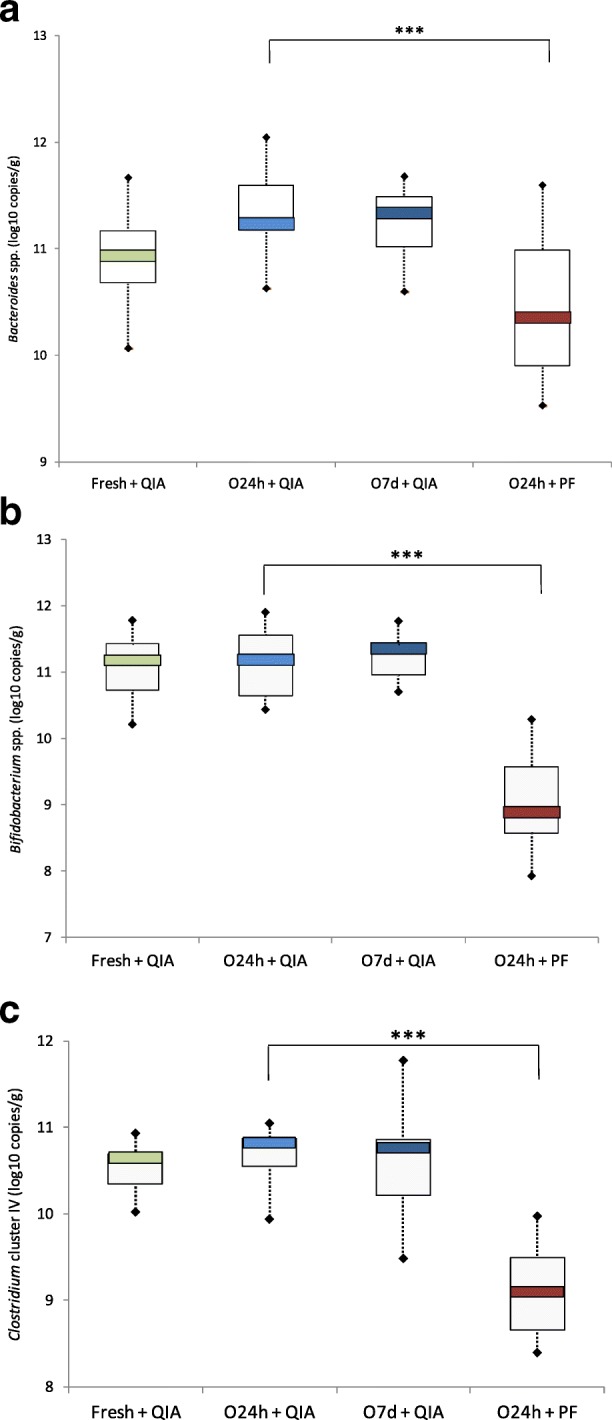
Log base 10 of gene copy number per gram of feces of *Bacteroides* spp. **a**, *Bifidobacterium* spp. **b** and *Clostridium* cluster IV (**c**) quantified by real-time PCR for the different collection and DNA extraction kits (***P < 0.001)

*Bacteroides* spp., *Bifidobacterium* spp. and *Clostridium* cluster IV were selected to confirm our results observed by the Illumina MiSeq sequencing. There were no differences in bacterial amounts among the collection/storage conditions. However, the quantification of *Bacteroides* (paired t-test; *P* < 0.001), *Bifidobacterium* (paired t-test; *P* < 0.001) and *Clostridium* cluster IV (paired t-test; *P* < 0.001) groups revealed significantly lower amounts in samples extracted by the PF compared to QIA, as shown in Fig. 5.

## Discussion

In this study, based on 14 fecal samples collected from healthy individuals (7 males, 7 females) we examined the performance of the OMNIgene•GUT kit in terms of bacterial DNA quantity and quality as an alternative to the collection of fresh human feces accounting for the impact of two different bacterial DNA extraction methods. To summarize our results, as a primary step we showed that read counts did not differ among all four comparisons (between collection and between DNA extraction methods). Furthermore, DNA concentration, purity and Shannon diversity index did not show differences between Fresh and OMNIgene•GUT kit.

The phylum relative abundance showed statistically significant differences in Bacteroidetes, Actinobacteria and Cyanobacteria between Fresh and O24h, but not between Fresh and O7d nor between O24h and O7d. This very specific effect could be explained by our limited sample size (*N* = 14) in combination with the number of tests performed (15 tests). When we increased the taxonomic resolution for subsequent analysis, this difference disappeared. The PCA analysis (Fig. 4) showed no differences between Fresh, O24h and O7d in terms of general microbial composition at the genus level. Furthermore when we validated our results, using qPCR of bacterial species representative of three tested phyla (see methods section) we confirmed that there were no differences (here at the species level) between the Fresh, O24h and O7d storage methods.

We determined the reliability of the OMNIgene•GUT kit accounting for two commonly used DNA extraction methods, which showed significant differences in terms of DNA quantity, quality, bacterial diversity and relative abundance. Revealing that the choice of DNA extraction method influences the gut microbiome profile. We discovered that by combining the OMNIgene•GUT kit and QIA extraction method we maximized the information content. However, we do not have objective reason why one method would work less efficient from the other.

Studies on the overall relative abundance and diversity of bacterial communities in fecal samples stored at room temperature have shown controversial results. Gorzelak et al. (2015) reported alterations in terms of bacterial taxa abundance and diversity in fecal samples stored at room temperature. These alterations might result from lack of nucleic acid stabilizer [22]. Additionally, the homogenization procedure showed an effect on the variability in gut microbiome data [22]. Of note, the OMNIgene•GUT kit was not included in those studies; this kit includes a homogenization step at the point of collection and contains microbial growth stabilizer.

The two other studies, Tedjo et al. (2015) and Dominianni et al. (2014) [23, 24], revealed no significant changes in microbiome structure and relative abundance. The OMNIgene•GUT kit was not included in those studies. Research by Tedjo et al. (2015) suggested that storage up to 24 h at room temperature did not affect the fecal microbial composition compared to direct freezing (-80 °C) of samples from healthy individuals (*N* = 10) and people with gastrointestinal disorders (*N* = 22). Importantly, they recommended applying a single storage method within a study to prevent potential bias in the results. Dominianni et al. (2015) did not find significant differences in overall microbial structure and relative abundance of storage at room temperature for 3 days compared to samples of healthy individuals (*N* = 3) immediately frozen at -80 °C. However, they acknowledged that a larger cohort could show variation.

As summarized in Table 4, several studies have investigated the impact of room temperature storage on microbiome composition, including the OMNIgene•GUT kit. Choo et al. (2015) reported no significant differences in Shannon diversity index (in agreement with our results), an increase of Proteobacteria relative abundance (we observed no changes) and no significant differences of Actinobacteria, Firmicutes and Bacteroidetes taxa for OMNIgene•GUT samples compared to frozen control (-80 °C). We observed significant differences in relative abundance of these two phyla, lower in Actinobacteria and higher in Bacteroidetes for O24h, and no differences for O7d when compared to Fresh. Choo et al. results are partially in line with our findings and this could be due to using different control groups (Choo et al. -80 °C and our 4 °C). However, Choo et al. showed that different sample storage conditions (-80 °C vs 4 °C) do not seem to have a significant impact on the microbial composition [25]. Another way to explain the abundance differences observed between our and Choo et al. results may be the use of different DNA extraction methods, which, as we could observe in the present study, had a significant impact on microbiome composition. Additionally, their results were based on a single individual while we investigated 14 participants.

**Table 4:** Comparison of the microbiome storage studies carried out since 2014

Number of individuals	Time of RT storage	Design	DNA extraction method	Techniques	Conclusion	Ref.
1	72 h	Comparison of OMNIgene·GUT/ RNA later/ Tris-EDTA buffer storage methods	MoBio Powelyser Powersoil DNA Isolation Kit	Illumina MiSeq 16S rRNA	Least alteration from OMNIgene·GUT	[25]
3	3 days	Comparison of RNAlater storage at RT for 3 days vs. storage at -80 °C	MO BIO Powelyser Powersoil DNA isolation kit	454 sequencing of 16S rRNA	RNAlater tend to show lower diversity and purity	[24]
18 (IBS-IBD patients + controls)	24 h	Comparison between storage at RT for 24 h/ storage at + 4 °C for 24 h/ storage at -20 °C for one week vs. storage at -80 °C	PSP lysis buffer+ beat-beating+ PSP Spin stool kit	454 sequencing of 16S RNA + qPCR on *Methanobrevibacter smithii*	No significant differences between the storage at RT for 24 h, storage at 4 °C for 24 h and storage at -20 °C for one week.	[23]
4	15 or 30 min	Comparison between storage at RT for 15 min vs. 30 min vs. no buffer	Qiagen stool Mini kit+ Bead-beating	qPCR	Fecal samples should be frozen within 15 min counting from collection.	[22]
41 (19 elderly + 22 infants)	7 or 14 days	Storage at RT within OMNIgene·GUT kit for 7 or 14 days vs. fresh samples	RBB	Ilumina MiSeq + PicoGreen	OMNIgene·GUT kit did not significantly impact microbiota composition and diversity in elderly datasets after 7d of storage. It can be used instead of fresh method.	[13]
14	24 h and 7 days	Comparison of storage at RT within OMNIgene·GUT for 24 h or 7 days vs freezing + comparison of two DNA extraction method	MO BIO Power Fecal DNA Isolation Kit vs. Qiagen QIAmp DNA Stool Mini kit+ bead-beating	Illumina MiSeq 16S RNA + qPCR on *Bacteroides* spp., *Bifidobacterium* spp. and *Clostriduim* cluster IV	Significant influence of DNA extraction method + no influence of storage within OMNIgene·GUT and between OMNIgene·GUT vs. Fresh in terms of microbial diversity and quantity	Our study

*Number of individuals* number of subjects used in the reference/our study, *time of RT storage* time of the storage of reference/our samples at room temperature (RT), *Design* short description of the tested aim, *DNA extraction method* method used for DNA extraction of fecal samples, *Techniques* name of the method used for microbial analysis, *Conclusion* the main point referring storage method, *Ref*. references where the method is described, *EDTA* ethylenediamine tetraacetic acid, *IBS* Irritable bowel syndrome, *IBD* inflammatory bowel disease, *qPCR* quantitative polimerase chain reaction, *RBB* repeat bead beating

Song et al. (2016) showed that storage of fecal samples (*N* = 15) using the OMNIgene•GUT kit for up to 8 weeks did not affect fecal microbial community structure. They recommend this kit to be used for microbiome studies including long-term studies, which is in concordance with our findings.

We investigated two different DNA extraction methods which yielded different microbial DNA concentrations and help explain the differences in microbiome composition observed between studies. Previous studies [26, 27] have also shown differences in terms of microbial diversity and abundance among different bacterial DNA extraction methods. Larsen et al. (2015) showed that the QIAamp DNA Stool Mini Kit (Qiagen, Valencia, CA) performed better than the DNeasy Blood and Tissue Kit (Qiagen, Valencia, CA) in terms of downstream analysis of fish gut microbiota. Peng et al. (2013) demonstrated the impact of 5 commercial DNA extraction methods on gut microbiota analysis. Similar patterns of bacterial communities were found in four out of five commercial kits (*N* = 1); an exception was the MO BIO method (UltraClean™ Fecal DNA Kit; MO BIO, USA). The MO BIO kit showed lower microbial diversity in the work of Peng [26], which is in concordance with our results on the use of PowerFecal® DNA Isolation Kit (MO BIO Laboratories, Inc.). Moreover, changes in the Firmicutes to Bacteroidetes ratio (F:B ratio) have been reported in the literature in patients with irritable bowel syndrome (IBS), obesity, and autism spectrum disorder (ASD) [28], and have been suggested as a potential biomarker. Our results indicate that the DNA extraction method has a strong effect on F:B ratio (PF 1:1 ratio; QIA 3.4:1 ratio) (Fig. 3). Therefore, the DNA extraction method has to be taken into account when comparing microbiome analysis across different sites (as well as a collection, storage, transport and 16S sequencing methods). Based on our results, the QIA method appears to be adequate for microbiome studies, since it showed higher DNA concentration, purity and bacterial diversity.

This study should be seen in the light of several strengths and limitations. The strengths of this study are the ability to compare different collection and storage methods while accounting for two different DNA extraction methods. We showed the results across two complementary techniques: qPCR and Illumina sequencing. The limitation of our study is the sample size. Larger sample sizes would further improve the generalizability of our results, increase statistical power and allow the detection of small differences between collection methods if any occur. We used 16S rRNA gene amplicon as an approach for microbiome analysis. Therefore, further investigations can take into account another common approach, metagenomics, in which bacterial structure and function are revealed based on whole genome sequencing.

## Conclusion

In this study, we highlighted the potential of using the OMNIgene•GUT kit for collection and storage at ambient temperature, which is convenient for studies aiming to collect large samples by giving participants the possibility to send samples by post. Our results underscore the importance of the choice of a DNA extraction method for the proper human gut microbial representation.

## Additional files

Additional file 1: **Figure S1.** Experimental design. Faecal samples were collected from 14 healthy participants in triplicates. Afterwards, the samples were stored (i) at 4 °C straight after collection and processed within 24 h (Fresh), (ii) at RT using the OMNIgene•GUT kit for 24 h (O24h) or (iii) for seven days (O7d) and then processed. (DOCX 94 kb)

Additional file 2: **Figure S2.** Experimental design. DNA from faecal samples was extracted using QIA and PF methods. DNA from Fresh, O24h and O7d samples was extracted using QIA method; DNA from the O24h samples was also extracted using PF method. (DOCX 76 kb)

Additional file 3: **Table S1.** Read counts information per samples collected and stored by three different collection/storage methods (Fresh, O24h, O7d) and extracted using two different procedures (PF, QIA). **Table S2.** Comparison of the read counts per samples between three different collection/storage methods (Fresh, O24h, O7d) and two different DNA extraction procedures (PF, QIA). Paired sample t-test showed no statistical differences between any of the groups in terms of read count. (XLSX 15 kb)

## Abbreviations

16S: 16S rRNA gene
ASD: Autism spectrum disorder
bp: base pair
dNTP: Deoxynucleoside triphosphate
DSMZ: Deutsche Sammlung von Mikroorganismen und Zellkulturen
IBS: Irritable bowel syndrome
mM: millimolar
ng: nanogram
NGS: Next generation sequencing
OTU: Operational taxonomy unit
PCA: Principal component analysis
PCR: Polymerase chain reaction
PEAR: Paired-End reAd mergeR
PF: PowerFecal® DNA Isolation Kit
pM: picomolar
PyNAST: Python Nearest Alignment Space Termination
QIA: QIAamp DNA Mini Kit
QIIME: Quantitative Insights into Microbial Ecology
qPCR: real-time PCR
rDNA: ribosomal DNA
RDP: Ribosomal database project
rRNA: ribosomal RNA
RT: Room temperature
spp.: species plural
μL: microlitre
μM: micromolar
