# Supplementary figures and images for: Reliability of a participant-friendly fecal collection method for microbiome analyses: a step towards large sample size investigation
Source: BMC Microbiol. 2018 Sep 6;18:110. doi: 10.1186/s12866-018-1249-x (PMC6127955; doi:10.1186/s12866-018-1249-x)

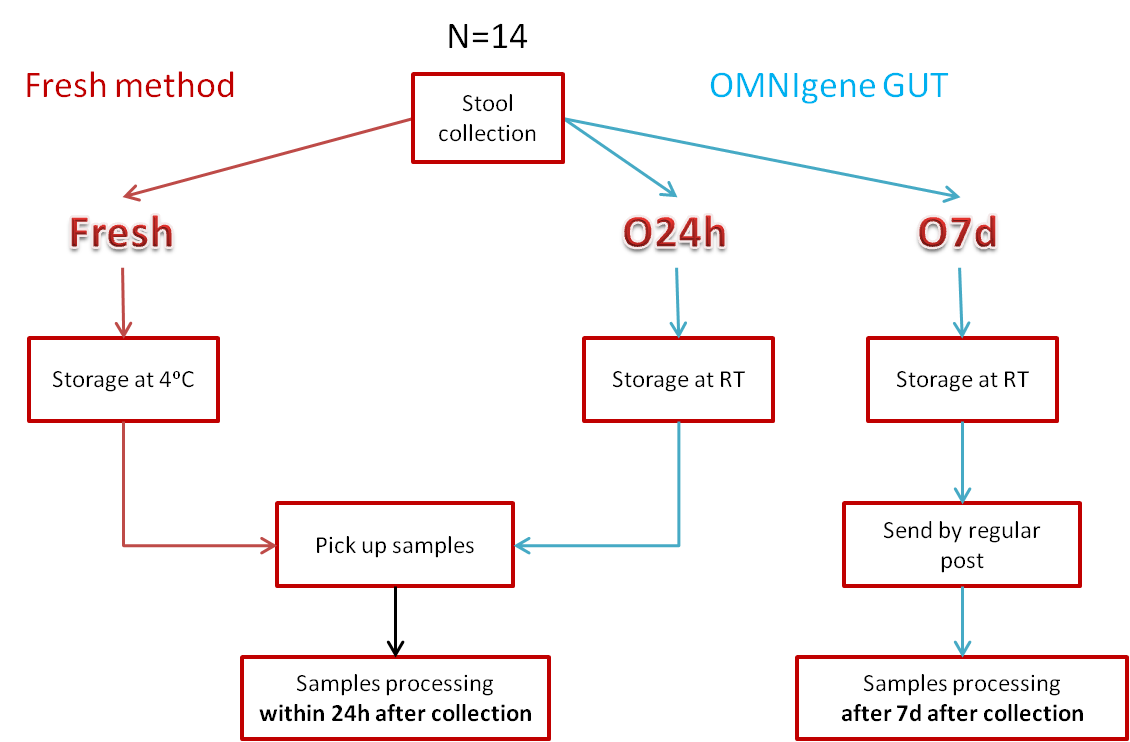

Supplement: Supplementary file 1 — Figure S1. Experimental design. Faecal samples were collected from 14 healthy participants in triplicates. Afterwards, the samples were stored (i) at 4 °C straight after collection and processed within 24 h (Fresh), (ii) at RT using the OMNIgene•GUT kit for 24 h (O24h) or (iii) for seven days (O7d) and then processed. (DOCX 94 kb) [file 12866_2018_1249_MOESM1_ESM.docx]

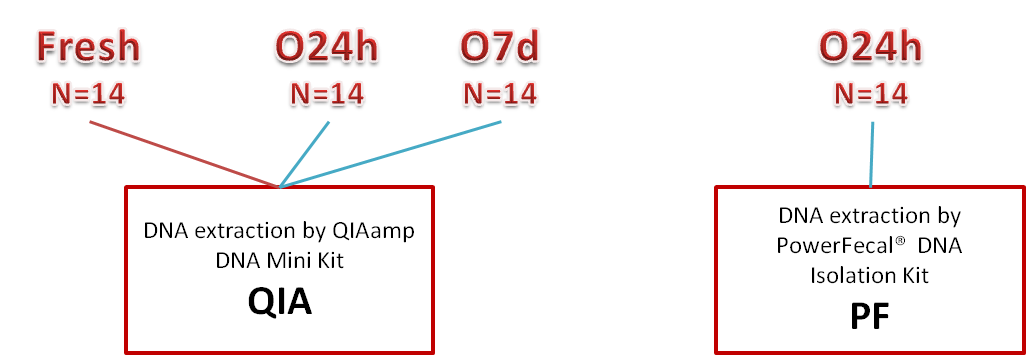

Supplement: Supplementary file 2 — Figure S2. Experimental design. DNA from faecal samples was extracted using QIA and PF methods. DNA from Fresh, O24h and O7d samples was extracted using QIA method; DNA from the O24h samples was also extracted using PF method. (DOCX 76 kb) [file 12866_2018_1249_MOESM2_ESM.docx]
